# DNA methylation-associated dysregulation of transfer RNA expression in human cancer

**DOI:** 10.1186/s12943-022-01532-w

**Published:** 2022-02-12

**Authors:** Margalida Rosselló-Tortella, Alberto Bueno-Costa, Laura Martínez-Verbo, Lorea Villanueva, Manel Esteller

**Affiliations:** 1 Cancer Epigenetics Group, Josep Carreras Leukaemia Research Institute (IJC), 08916 Badalona, Catalonia Spain; 2 Germans Trias i Pujol Health Science Research Institute (IGTP), 08916 Badalona, Catalonia Spain; 3 Centro de Investigacion Biomedica en Red Cancer (CIBERONC), 28029 Madrid, Spain; 4 Institució Catalana de Recerca i Estudis Avançats (ICREA), 08010 Barcelona, Catalonia Spain; 5 Physiological Sciences Department, School of Medicine and Health Sciences, University of Barcelona (UB), 08907 Barcelona, Catalonia Spain; 6 Josep Carreras Leukaemia Research Institute (IJC), Carretera de Can Ruti, Camí de les Escoles s/n, 08916 Badalona, Barcelona, Catalonia Spain

The human cytoplasmatic pool of tRNA for the 20 proteinogenic amino acids and selenocysteine is composed of 48 isoacceptor families –those tRNA with different anticodons– divided into 253 different isodecoder species –those tRNAs that share the same anticodon but present sequence variations in other positions [1, 2]. All these molecules cooperate to translate the genetic information encoded in mRNA to enable protein synthesis. For many years, tRNAs have been considered as housekeeping molecules without any additional regulatory function, but compelling recent evidence of the intricacy of tRNA biology have proven that this initial misconception was far from reality. In fact, tRNAs actively engage in protein synthesis regulation and in additional molecular processes that are unrelated to translation, like apoptosis prevention and the generation of small derivative non-coding RNAs that perform further cellular functions [2].

The advances in high-throughput sequencing technologies have demonstrated that tRNAs are differentially transcribed and expressed among tissues [3]. Such intricacy in tRNA expression regulation cannot be exclusively explained by the global modulation of the RNA polymerase III (RNAPIII) transcriptional activity. Thus, the definition of additional mechanisms is needed to clarify such precision in tRNA expression control. Nucleosome positioning [4], histone modifications [5], and the transcription of neighboring genes by the RNA polymerase II (RNAPII) [6] are known to influence RNAPIII transcription, and therefore may contribute to this specificity.

The importance of tRNAs in cell physiology is emphasized by the discovery that these molecules and their derived fragments are distorted in pathological processes, including cancer [7]. Cancer-associated tRNA deregulation has been overlooked for many years, but now these molecules have been directly connected to tumorigenesis and are postulated to undertake active roles in this process [7]. Tumor-related alterations in tRNA occur throughout their bioprocessing, from transcription to nucleoside modification and cleavage [7]. For instance, the altered expression of tRNA modifier enzymes reshapes the tRNA modification landscape and contributes to the disease [8]. The increased abundance of tRNAs in tumoral cells was first esteemed to be a natural outcome of the increased protein synthesis rate that is intrinsic of highly proliferative cells. This is in part due to the number of oncogenic and tumor suppressive pathways that promote RNAPIII activity and lead to a global boost in tRNA expression [7]. tRNA overexpression in tumoral cells is extensive to all tumor types, but not all tRNA species are equally altered [9]. The mechanisms that orchestrate this specific control of tRNA expression in cancer cells are not completely understood. These changes in the tRNA pool composition facilitate the translation of transcripts with certain codons [10] and alter the abundance of small tRNA-derived fragments to foster tumorigenesis [11]. This is reflected in the differential patient survival or drug response that arise from alterations in tRNAs, their modifier enzymes and in their derived fragments [8, 12].

Alterations in DNA methylation constitute a frequent mechanism by which tumor cells inactivate the expression of many coding and non-coding genes to acquire their malignant features [13]. This epigenetic mechanism is reported to hamper the binding of the RNAPIII general transcription factor 3 C (TFIIIC) [14] to DNA and prevent tRNA transcription *in vitro* [15]. However, the real degree of DNA methylation involvement in tRNA expression regulation remains unexplored. Herein, we show for the first time that variations in DNA methylation at tDNA influence tRNA expression and may account for the specific alterations in tRNA levels observed in cancer cells that can enhance the progression of the disease.

## Results and Discussion

### Shifts in the DNA methylation patterns of tDNAs occur in human tumorigenesis

To identify DNA methylation defects in tDNA, we retrieved the Infinium HumanMethylation450 (HM450) methylation microarray probes’ identity that mapped into the loci corresponding to the 416 high confidence tDNA that are found in the human nuclear genome [1]. 138 tDNAs were represented in the HM450 microarray, and 95 of them were located further than 2 kb from RNAPII-transcribed genes (Supplementary Figure S1A). We established this exclusion criterion because the interferences mediated by elongating RNAPII on RNAPIII transcription of neighboring genes could mask the effects that DNA methylation would induce on tRNA expression. We identified 71 tDNA genes that were represented by at least one CpG in the HM450 microarray whose methylation status could be efficiently interrogated (Supplementary Figure S1A).

**Fig. 1:**
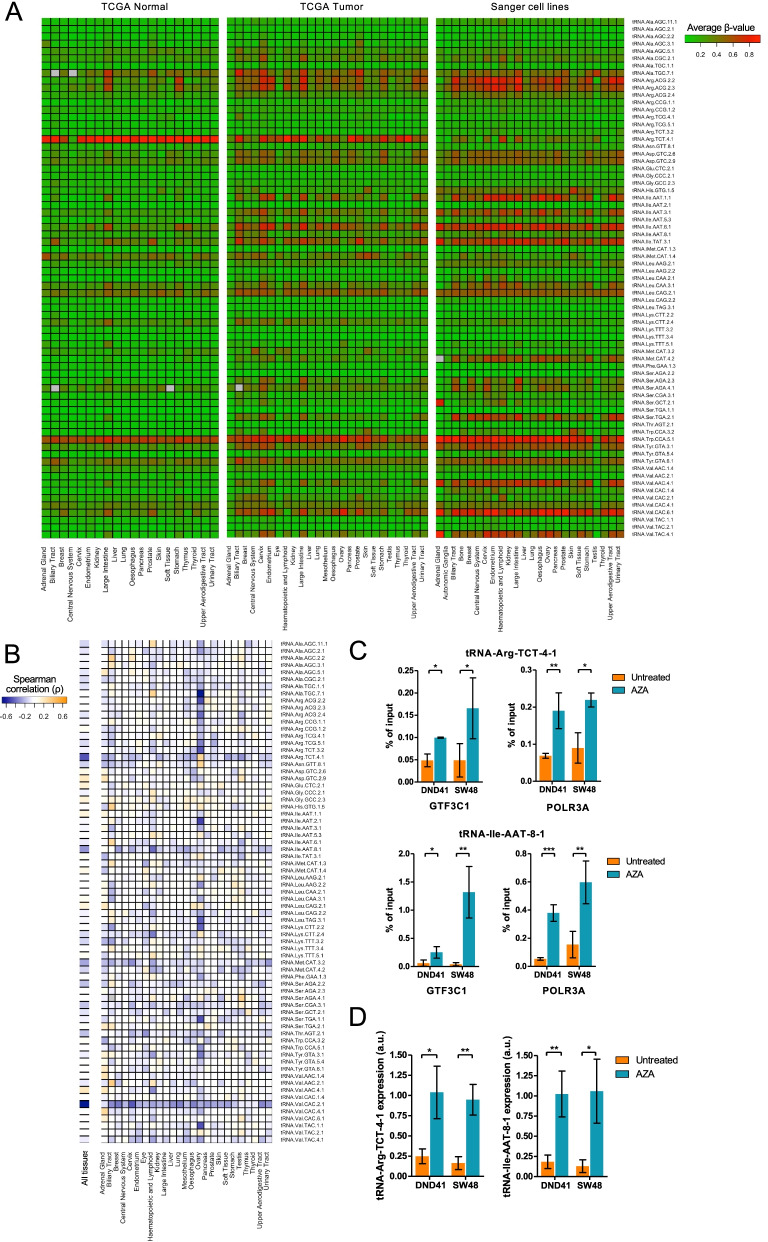
Tumor-associated changes in tDNA methylation promote differences in tRNA expression. **A** Heatmaps showing the average β-value for each tDNA in the different tissues from TCGA set of normal (*left*) and tumoral (*middle*) samples and in cancer cell lines (*right*). β-values span from 0 (green, unmethylated) to 1 (red, hypermethylated). Grey indicates missing values. **B** Heatmap showing the Spearman’s ρ of the correlation between tDNA gene methylation and the expression of their cognate tRNA in all TCGA tumor samples (*left*) and separated by tissue of origin (*right*). Yellow and blue denote positive and negative correlation, respectively. **C** ChIP-qPCR experiments indicate an increased binding of GTF3C1 (*left*) and POLR3A (*right*) to tRNA-Arg-TCT-4-1 (*top*) and tRNA-Ile-AAT-8-1 (*below*) genes upon treatment with 5’-azacytidine (AZA) in DND41 and SW48. Data represent the mean ± SD of three biological replicates analyzed using an unpaired two-tailed Student’s t-test. * *p* < 0.05; ** *p* < 0.01; *** *p* < 0.001. **D** qRT-PCR shows a recovery of tRNA-Arg-TCT-4-1 (*left*) and tRNA-Ile-AAT-8-1 (*right*) expression in DND41 and SW48 cell lines after the use of AZA. All qRT-PCR data represent the mean ± SD of biological triplicates analyzed using the unpaired two-tailed Student’s t-test. * *p* < 0.05; ** *p* < 0.01.

The data mining of HM450 microarray-derived data of thousands of samples from various cancer types and normal tissues available at The Cancer Genome Atlas (TCGA) and of a panel encompassing approximately 1000 cell lines revealed global differences in tDNA methylation in TCGA primary tumors and cancer cell lines compared to TCGA normal samples, mostly tending towards hypermethylation in malignant samples (Supplementary Figure S1B). Specifically, 60 of the 71 tDNA presented statistically significant differences between normal and tumoral TCGA sets of samples (Supplementary Figure S1B). The separation of TCGA and cell line samples according to their origin showed that the different normal and tumoral tissues also presented variations in tDNA methylation levels according to the cancer type (Fig. 1A). These findings indicate that tumorigenesis involves epigenetic lesions in tDNA genes that are characteristic among different tissues.

### tDNA methylation is associated with reduced tRNA expression in cancer cells

The differences in tDNA methylation status between normal and malignant samples and among tissues prompted us to investigate whether this phenomenon could drive cancer-associated and tissue-specific variations in the expression of specific tRNAs. To address this, we evaluated the *in silico* correlation between tDNA methylation and the expression of their cognate tRNA using a publicly available dataset of tRNA expression in TCGA samples [9]. Three tRNAs presented a statistically significant Spearman’s correlation coefficient (*ρ* ≤ -0.2) when considering all the samples: tRNA-Arg-TCT-4-1, tRNA-Ile-AAT-8-1, and tRNA-Val-CAC-2-1 (Fig. 1B). Since tDNA methylation presented differences depending on the origin of the sample (Fig. 1 A), we also investigated if the association between tDNA methylation and tRNA expression was dependent on the tissue (Fig. 1B). From the total 1775 computed Spearman’s correlations, 1073 revealed a negative association between tDNA methylation and tRNA expression (Fig. 1B). 114 of the computed correlations displayed a significant anticorrelation (*ρ* ≤ -0.2; Supplementary Figure S2A). tRNA-Arg-TCT-4-1, tRNA-Ile-AAT-8-1, and tRNA-Val-CAC-2-1 were those tRNAs with the highest frequency of tissues displaying a negative association between tDNA methylation and tRNA expression (Supplementary Figure S2B). This negative correlation between tDNA methylation and tRNA levels in TCGA suggests a plausible link between DNA methylation and tRNA expression.

Following the above described findings, we aimed to analyze in more detail the relationship between DNA methylation and tRNA expression by providing additional experimental evidence of the epigenetic regulation of two tDNA whose methylation is susceptible to impact tRNA expression according to our analysis: tRNA-Arg-TCT-4-1 and tRNA-Ile-AAT-8-1. tRNA-Arg-TCT-4-1 is methylated in most normal tissues, whereas tumoral samples show hypomethylation events when compared to their matched healthy counterparts. (Figure 1A). Conversely, tRNA-Ile-AAT-8-1 is unmethylated in normal tissues and hypermethylated in cancer (Fig. 1A). We selected two cell lines that were hypermethylated for both genes according to the HM450 microarray data: DND41 and SW48 (Supplementary Figure S3A). Both tDNAs are represented in the HM450 microarray with a single CpG that is located within the internal promoter of the gene –the conserved A and B boxes where TFIIIC binds to recruit the RNAPIII machinery [2]. We confirmed by bisulfite genomic sequencing of multiple clones the methylation status of the two selected tDNA (Supplementary Methods) in these cell lines and obtained a comprehensive view of the whole methylation landscape for each of them (Supplementary Figure S3B). DNA demethylation upon the use of the drug 5’-azacytidine on these cell lines increased the binding of the subunit 1 of TFIIIC (GTF3C1) and the subunit A of RNAPIII (POLR3A) to the two tDNA genes as measured by chromatin immunoprecipitation (ChIP)-qPCR (Supplementary Methods), indicating that DNA methylation prevents the binding of the tDNA-associated transcriptional machinery to the internal promoter of the tDNAs (Fig. 1 C). Moreover, the treatment with the demethylating agent 5’-azacytidine also restored the expression of both tRNAs in the two interrogated hypermethylated cell lines (Fig. 1D) as determined by qRT-PCR (Supplementary Methods), confirming that DNA methylation can repress RNAPIII-mediated gene expression. The use of the DNA methylation inhibitor, and its associated restoration of tRNA expression upon demethylation, reduced cell growth (Supplementary Figure S4A) and induced apoptosis (Supplementary Figure S4B). Thus, our *in vitro* results showing an increase in tRNA expression in hypermethylated cell lines upon DNA demethylation are in accordance with the negative relationship between tDNA methylation and tRNA expression that arose from the *in silico* analysis in TCGA (Fig. 1B).

### tDNA methylation can predict the patients’ clinical outcome in TCGA cohorts

**Fig. 2:**
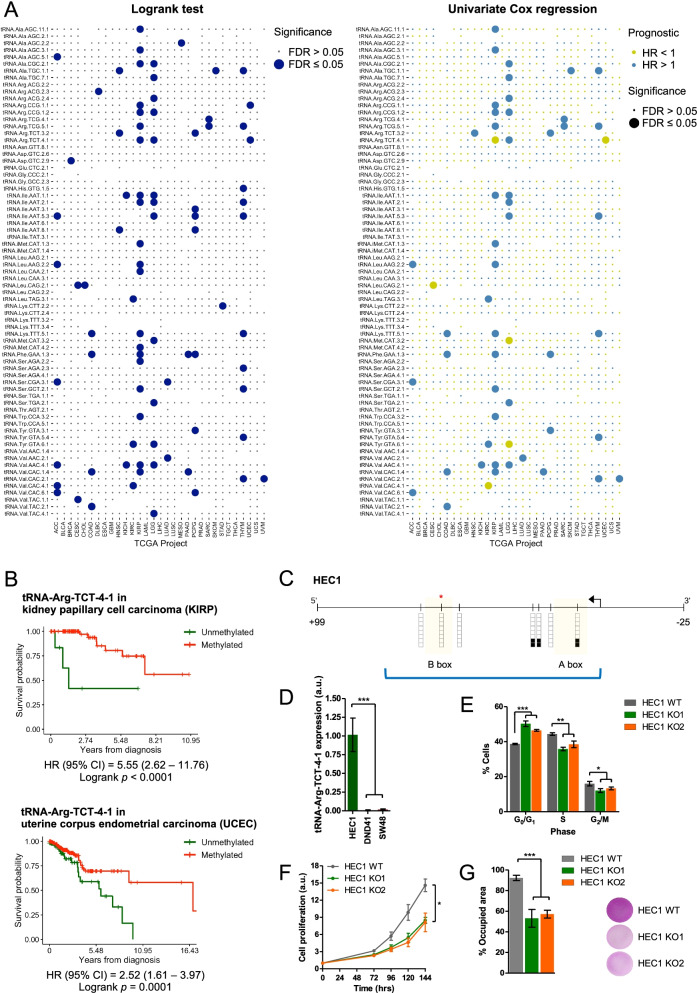
tDNA methylation is associated with different overall survival in TCGA cohorts. **A** Dot plots summarizing the logrank tests (*left*) and univariate Cox regression models (*right*) used to compare the overall survival of patients according to methylation status of the 71 tDNAs. Cases that are significantly associated with different prognosis are represented in large-sized bullets. Yellow and blue represent favorable and unfavorable prognosis according to univariate Cox regression analyses (*right*), respectively. HR, hazard ratio. **B** Kaplan-Meier curves show that tRNA-Arg-TCT-4-1 hypomethylation is associated with a shorter overall survival in KIRP (*top*) and UCEC (*below*) TCGA cohorts. HR, hazard ratio; CI, confidence interval. *p*-values correspond to logrank tests. **C** Bisulfite genomic sequencing confirms tRNA-Arg-TCT-4-1 hypomethylation in HEC1 cell line. The tDNA sequence is indicated with a blue bracket. The orange rectangles correspond to the A and B boxes of the tDNA. The TSS is marked with a black arrow. CpG dinucleotides are represented as short vertical lines, and their methylation status is denoted with black (methylated) or white (unmethylated) squares. The CpG represented in the HM450 microarray is marked with a red asterisk. **D** qRT-PCR exposes higher tRNA-Arg-TCT-4-1 levels in HEC1 compared to DND41 and SW48 cells. Data represent the mean ± SD of biological triplicates analyzed by an unpaired two-tailed Student’s t-test. *** *p* < 0.001. **E** Cell cycle analysis reveals an accumulation of tRNA-Arg-TCT-4-1 knockout HEC1 cells in G0/G1 phase. Data shown represent the mean ± SD of biological triplicates analyzed by unpaired two-tailed Student’s t-test. * *p* < 0.05. **F** SRB assay shows a reduced growth of HEC1 knockout cells. Data at each time points are the mean ± SD of four biological replicates. Statistical differences were determined using an unpaired two-tailed Student’s t-test at the 144 h final time point. * *p* < 0.05. **G** Transwell assay shows a reduced migration of tRNA-Arg-TCT-4-1-silenced HEC1 cells. Data represent the mean ± SD of biological triplicates analyzed by an unpaired two-tailed Student’s t-test. *** *p* < 0.001. Representative images of the Transwell insert membranes are shown.

Apart from modulating gene expression to promote the malignant transformation of the cell and facilitate cancer progression, tumor-specific alterations in DNA methylation can be translated into the clinical practice as potential biomarkers to predict the evolution of the disease [13]. The expression of some tRNA has also been associated to different clinical outcomes of cancer patients [9, 12]. For this, because we observed that the changes in tDNA methylation that occur in tumorigenesis modulate tRNA expression, we sought to evaluate if these events had any impact on the outcome of cancer patients and therefore could serve as predictive biomarkers for this disease. To address this hypothesis, we data mined the clinical information from 31 TCGA projects to assess if differential tDNA methylation could inform patients’ survival. We identified 86 cases in which the methylation status of a specific tDNA was significantly associated with differences in patients’ overall survival (Fig. 2A). 56 of these events were confirmed to be prognostic predictive factors for the disease according to univariate Cox regression analyses (Fig. 2 A, Supplementary Table S1). Since the tRNA pool composition is reported to vary among tissues to match the specific cellular needs and permit the expression of different sets of proteins [16], whether each differential tDNA methylation event results in an increased or a reduced risk of the disease should be evaluated individually for every gene and tumor (Fig. 2 A, Supplementary Table S1).

### The silencing of tRNA-Arg-TCT-4-1 reduced endometrial cancer cell growth

Our analyses identified tRNA-Arg-TCT-4-1 as a promising candidate to participate in tumorigenesis. Interestingly, high levels of tRNA-Arg-TCT-4-1 are linked to a proliferative status of the cell [17], which fits with the cancer-associated demethylation of this tDNA in comparison with the methylated status of the normal tissues (Fig. 1 and Supplementary Figure S1A) and its resulting overexpression in 12 cancer types represented in TCGA [9]. Importantly, tRNA-Arg-TCT-4-1 demethylation in kidney renal papillary cell carcinoma (KIRP) and in uterine corpus endometrial carcinoma (UCEC) cohorts of TCGA primary tumors is associated to a shorter overall survival (Fig. 2B). This hypomethylation change in KIRP and UCEC TCGA cohorts accounts for an 8% and 29% of cases, respectively (Supplementary Figure S5A). In both cases, the hypomethylation of the gene was associated to an increased expression of the tRNA (Supplementary Figure S5B). This is consistent with the overexpression of this tRNA in a subgroup of tumoral samples compared to the normal matched tissue [9] that is methylated (Fig. 1 A and Supplementary Figure S1A). The higher percentage of cases presenting tRNA-Arg-TCT-4-1 demethylation in the UCEC cohort of primary tumors (Supplementary Figure S5A) prompted us to investigate the role of this tRNA in endometrial cancer and confirm the proposed oncogenic role of its demethylation. The prognosis for advanced forms of this disease is poor, and therapeutic options beyond surgery and first-line chemotherapy are limited [18]. Therefore, there is an urgent need to investigate this malignancy and describe biomarkers that predict its aggressiveness to spot high-risk patients that would need a more comprehensive vigilance or that would benefit from receiving adjuvant therapy after the surgical resection of the tumor [18].

First, we verified by bisulfite genomic sequencing of multiple clones the unmethylated status of tRNA-Arg-TCT-4-1 gene in the endometrial cancer cell line HEC1 (Fig. 2 C), as previously noted in the overall screening of cancer cell lines (Fig. 1 and Supplementary Figure S1B). We also confirmed that expression of this tRNA was higher in HEC1 than in the hypermethylated DND41 and SW48 cell lines (also derived from our *in silico* screening, Fig. 1and Supplementary Figures S1B and S3A) to support that tDNA hypermethylation represses tRNA expression (Fig. 2D). Changes in tRNA expression can drive tumorigenesis as they can confer advantages to the malignant cell to promote cancer progression and dissemination [7]. To further characterize the role of tRNA-Arg-TCT-4-1 in endometrial carcinoma, we eliminated this gene from HEC1 cell line using the CRISPR/Cas9 system (Supplementary Methods) to mimic the effects of its hypermethylation-associated silencing (Supplementary Figure S6A). This deletion was confirmed by genomic PCR and Sanger sequencing in two HEC1 knockout clones (Supplementary Figure S6B), which showed minimal tRNA-Arg-TCT-4-1 expression compared to the wild-type cell line (Supplementary Figure S6C). tRNA-Arg-TCT-4-1 CRISPR/Cas9-mediated deletion delayed the progression of the cell cycle, as seen by an accumulation of cells in the G0/G1 phase in detriment of the S and G2/M phases in these cells (Fig. 2E). This resulted in a reduced growth of HEC1 knockout cells compared to the wild-type cell line, as assessed by the SRB assay (Fig. 2 F). Additionally, tRNA-Arg-TCT-4-1 silencing also reduced cell migration in the two HEC1 knockout clones (Fig. 2G). Overall, our results indicate that tRNA-Arg-TCT-4-1 demethylation and increased expression exerts oncogenic-like roles in endometrial cancer by promoting cell growth, in agreement with the observed reduced overall survival of those cancer patients that undergo hypomethylation-associated activation of tRNA-Arg-TCT-4-1 (Fig. 2B and Supplementary Figure S5B).

## Conclusions

Tumoral cells present defects in tRNA biology at multiple levels. The altered expression of tRNA in cancer cells can support proliferation and drive tumorigenesis [7], but the causes and consequences of this deregulation are poorly understood. Together, our findings give insight on the contribution of DNA methylation to these changes in tRNA levels. Our study revealed that the malignant transformation of a cell entails epigenetic lesions in tDNA genes and provided for the first time a plausible connection between DNA methylation and tRNA expression. Most importantly from a clinical standpoint, these differential DNA methylation events can serve as predictive biomarkers for cancer patients’ prognosis, such as the case for the tRNA-Arg-TCT-4-1 demethylation-associated overexpression in endometrial carcinoma. These results support that tRNAs are active participants in tumorigenesis and that shifts in the DNA methylation patterns in their regulatory regions contribute to the altered profiles of tRNA expression observed in human cancer. However, much remains to be discovered about how their disruption affect cancer cell biology and its molecular pathways.

## Materials and method

Detailed materials and methods are available in the Supplementary Methods. The sequences of all primers used are provided in Supplementary Table S2.

## Supplementary Information

**Additional file 1: Figure S1. **Description of tDNA methylation using the HM450 microarray. (A) 416 high confidence tDNAs annotated in the GRCh37/hg19 human genome (top). 138 of them are included in the HM450 DNA methylation microarray. 95 of these 138 are located further than 2 kb from any other RNAPII-transcribed gene. The withdrawal of cross-reactive CpGs yielded the 71 different tDNAs that can be efficiently interrogated with this approach (below). The distribution by amino acid and anticodon of the 416 total tDNA genes (top) and those of the 71 that are represented in the HM450 DNA methylation microarray (below) are provided. (B) Average methylation of the 71 tDNA genes according to the HM450 DNA methylation microarray data in normal (left) and tumor (middle) TCGA samples and in cell lines (right). FDR-adjusted p-values correspond to the Mann-Whitney U-test used to compare the methylation average between TCGA normal and tumor samples. ns, not significant; * FDR < 0.05; ** FDR < 0.01; *** FDR < 0.001.

**Additional file 2: Figure S2. **The methylation status of tRNA-Arg-TCT-4-1, tRNA-Ile-AAT-8-1, and tRNA-Val-CAC-2-1 is associated with their expression levels in TCGA tumors. (A) Volcano plot representing the Spearman’s correlation coefficient (ρ) and -log10 FDR of each computed Spearman’s correlation from Figure 1B. Statistically significant associations are shown in blue. (B) Barplot summarizing the tissue frequency of all the statistically significant negative associations between methylation and expression from panel A.

**Additional file 3: Figure S3. **Bisulfite genomic sequencing confirms the methylation status of tRNA-Arg-TCT-4-1 andtRNA-Ile-AAT-8-1 according to the HM450 microarray data. (A) DNA methylation status of tRNA-Arg-TCT-4-1 (cg12798524) and tRNA-Ile-AAT-8-1 (cg21339923) in DND41 and SW48 according to the HM450 microarray data. b-values corresponding to single CpGs are shown for each tDNA. (B) Bisulfite genomic sequencing of tRNA-Arg-TCT-4-1 (top) and tRNA-Ile-AAT-8-1 (below) genes in DND41 and SW48 cell lines. The tDNA gene genomic sequence is indicated with a blue bracket. The orange rectangles correspond to the A and B boxes of the tDNA. The TSS is marked with a black arrow. CpG dinucleotides are represented as short vertical lines, and their methylation status is denoted with black (methylated) or white (unmethylated) squares. The CpG included in the HM450 microarray is marked with a red asterisk.

**Additional file 4: Figure S4. **Impact on growth and apoptosis of the DNA methylation inhibitor 5’-azacytidine(AZA) in DND41 and SW48 cancer cell lines. (A) MTT assay growth assay show a reduced growth of treated cells in both DND41 and SW48 cells lines upon the use of the demethylating agent. Data at each time points are the mean ± SD of four biological replicates. Statistical differences were determined using an unpaired two-tailed Student’s t-test at the 96 hours final time point. *** p < 0.001 (B) Apoptosis quantification by Annexin V incorporation reveals a significant percentage of cells affected by the treatment in DND41 and SW48 cell lines. Data shown represent the mean ± SD of four biological replicates analyzed by unpaired two-tailed Student’s t-test. * p < 0.05; *** p < 0.001.

**Additional file 5: Figure S5. **The demethylation of tRNA-Arg-TCT-4-1 gene is associated with an increased tRNA expression in KIRP and UCEC TCGA tumors. (A) Frequency of tRNA-Arg-TCT-4-1 hypomethylation in tumors derived from TCGA according to the tissue of origin. (B) tRNA-Arg-TCT-4-1 expression is higher in those KIRP (top) and UCEC (below) TCGA tumors where this gene is hypomethylated. Statistical differences in tRNA expression between groups of samples were determined using a two-sided Mann-Whitney U-test; *** *p* < 0.001. Samples were considered hypomethylated or hypermethylated when tRNA-Arg-TCT-4-1 β-value was lower or higher than 0.33, respectively.

**Additional file 6: Figure S6.**Generation of a tRNA-Arg-TCT-4-1 knockout model in HEC1 cell line using the CRISPR/Cas9 system. (A) Overview of the CRISPR/Cas9 system targeting tRNA-Arg-TCT-4-1 gene. Two different sgRNA (in green and orange) were simultaneously transfected to introduce a deletion of the gene of interest. (B) Genomic PCR using primers flanking tRNA-Arg-TCT-4-1 (black arrows in the overview representation) shows a deletion in two HEC1 knockout cells. (C) qRT-PCR confirms the silencing of tRNA-Arg-TCT-4-1 in the selected HEC1 knockout cells. Data correspond to the mean ± SD of three biological replicates analyzed using the unpaired two-tailed Student’s t-test. *** *p* < 0.001.

**Additional file 7:** Supplementary methods

**Additional file 8: Table S1. **Summary of the results obtained from the Univariate Cox regression analyses performed to compare TCGA patients’ prognosis according to their tDNA methylation levels. Blue-colored cells indicate statistical significance (FDR ≤ 0.05).

**Additional file 9: Table S2. **Detailed list of primers used. All sequences are provided from 5’ to 3’. For tRNA qRT-PCR primers, the commercial reference is provided.

## Data Availability

Not applicable.

## Abbreviations

tRNA: Transfer RNA
tDNA: tRNA gene
RNAPII: RNA polymerase II
RNAPIII: RNA polymerase III
HM450: HumanMethylation450
TFIIIC: RNAPIII general transcription factor
ChIP: Chromatin immunoprecipitation
GTF3C1: TFIIIC subunit 1
POLR3A: RNAPIII subunit A
TCGA: The Cancer Genome Atlas
KIRP: Kidney renal papillary cell carcinoma
UCEC: Uterine corpus endometrial carcinoma
