# Supplementary material for: DNA methylation-associated dysregulation of transfer RNA expression in human cancer
Source: Mol Cancer. 2022 Feb 12;21:48. doi: 10.1186/s12943-022-01532-w (PMC8840503; doi:10.1186/s12943-022-01532-w)
Supplement: Supplementary file 1 — Additional file 1: Figure S1. Description of tDNA methylation using the HM450 microarray. (A) 416 high confidence tDNAs annotated in the GRCh37/hg19 human genome (top). 138 of them are included in the HM450 DNA methylation microarray. 95 of these 138 are located further than 2 kb from any other RNAPII-transcribed gene. The withdrawal of cross-reactive CpGs yielded the 71 different tDNAs that can be efficiently interrogated with this approach (below). The distribution by amino acid and anticodon of the 416 total tDNA genes (top) and those of the 71 that are represented in the HM450 DNA methylation microarray (below) are provided. (B) Average methylation of the 71 tDNA genes according to the HM450 DNA methylation microarray data in normal (left) and tumor (middle) TCGA samples and in cell lines (right). FDR-adjusted p-values correspond to the Mann-Whitney U-test used to compare the methylation average between TCGA normal and tumor samples. ns, not significant; * FDR < 0.05; ** FDR < 0.01; *** FDR < 0.001. [file 12943_2022_1532_MOESM1_ESM.pptx]

## Slide 1
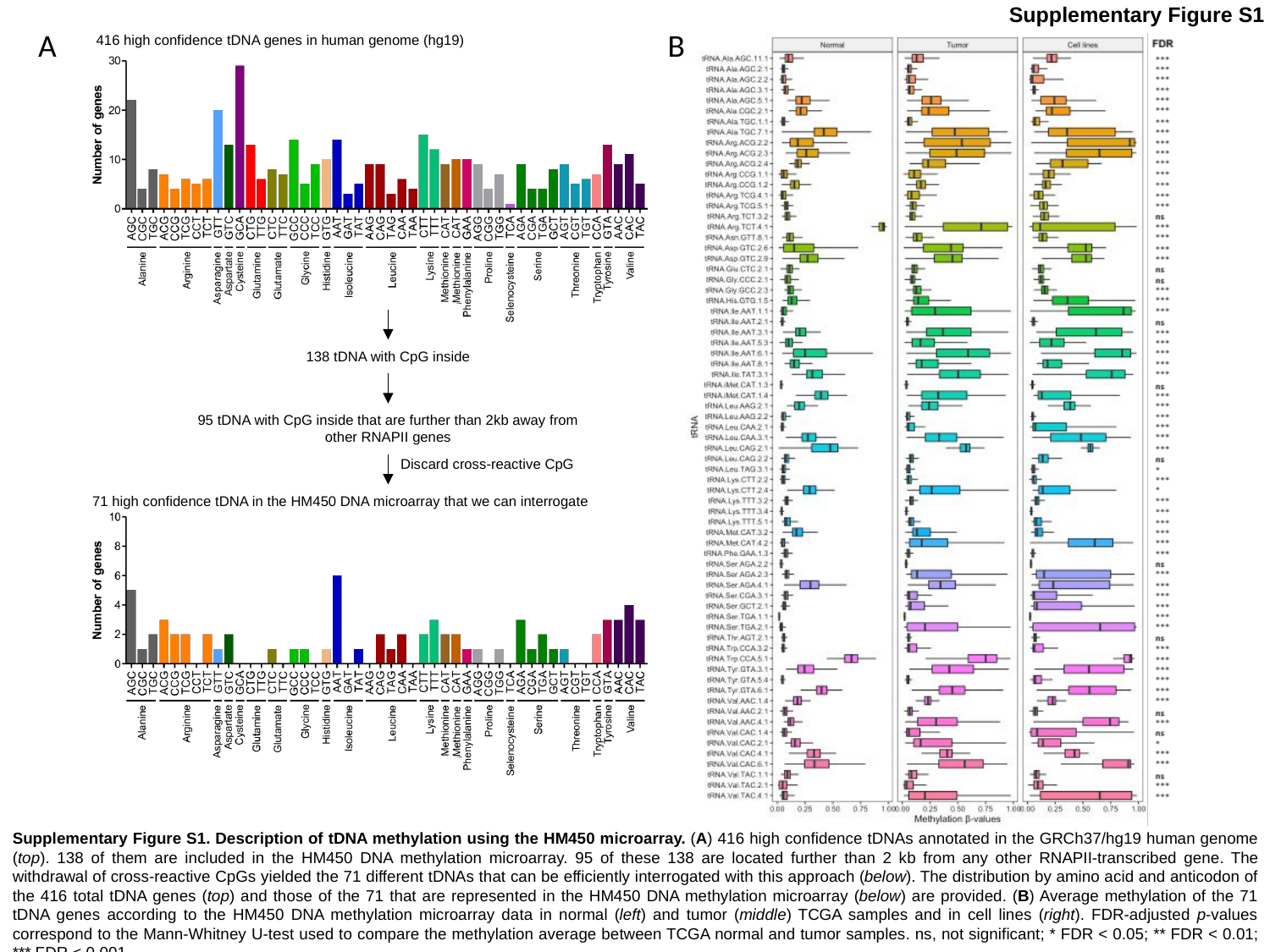

Supplementary Figure S1
A
B
416 high confidence tDNA genes in human genome (hg19)
95 tDNA with CpG inside that are further than 2kb away from other RNAPII genes
Discard cross-reactive CpG
138 tDNA with CpG inside
71 high confidence tDNA in the HM450 DNA microarray that we can interrogate
Supplementary Figure S1. Description of tDNA methylation using the HM450 microarray. (A) 416 high confidence tDNAs annotated in the GRCh37/hg19 human genome (top). 138 of them are included in the HM450 DNA methylation microarray. 95 of these 138 are located further than 2 kb from any other RNAPII-transcribed gene. The withdrawal of cross-reactive CpGs yielded the 71 different tDNAs that can be efficiently interrogated with this approach (below). The distribution by amino acid and anticodon of the 416 total tDNA genes (top) and those of the 71 that are represented in the HM450 DNA methylation microarray (below) are provided. (B) Average methylation of the 71 tDNA genes according to the HM450 DNA methylation microarray data in normal (left) and tumor (middle) TCGA samples and in cell lines (right). FDR-adjusted p-values correspond to the Mann-Whitney U-test used to compare the methylation average between TCGA normal and tumor samples. ns, not significant; * FDR < 0.05; ** FDR < 0.01; *** FDR < 0.001.
