# Supplementary material for: DNA methylation-associated dysregulation of transfer RNA expression in human cancer
Source: Mol Cancer. 2022 Feb 12;21:48. doi: 10.1186/s12943-022-01532-w (PMC8840503; doi:10.1186/s12943-022-01532-w)
Supplement: Supplementary file 2 — Additional file 2: Figure S2. The methylation status of tRNA-Arg-TCT-4-1, tRNA-Ile-AAT-8-1, and tRNA-Val-CAC-2-1 is associated with their expression levels in TCGA tumors. (A) Volcano plot representing the Spearman’s correlation coefficient (ρ) and -log10 FDR of each computed Spearman’s correlation from Figure 1B. Statistically significant associations are shown in blue. (B) Barplot summarizing the tissue frequency of all the statistically significant negative associations between methylation and expression from panel A. [file 12943_2022_1532_MOESM2_ESM.pptx]

## Slide 1
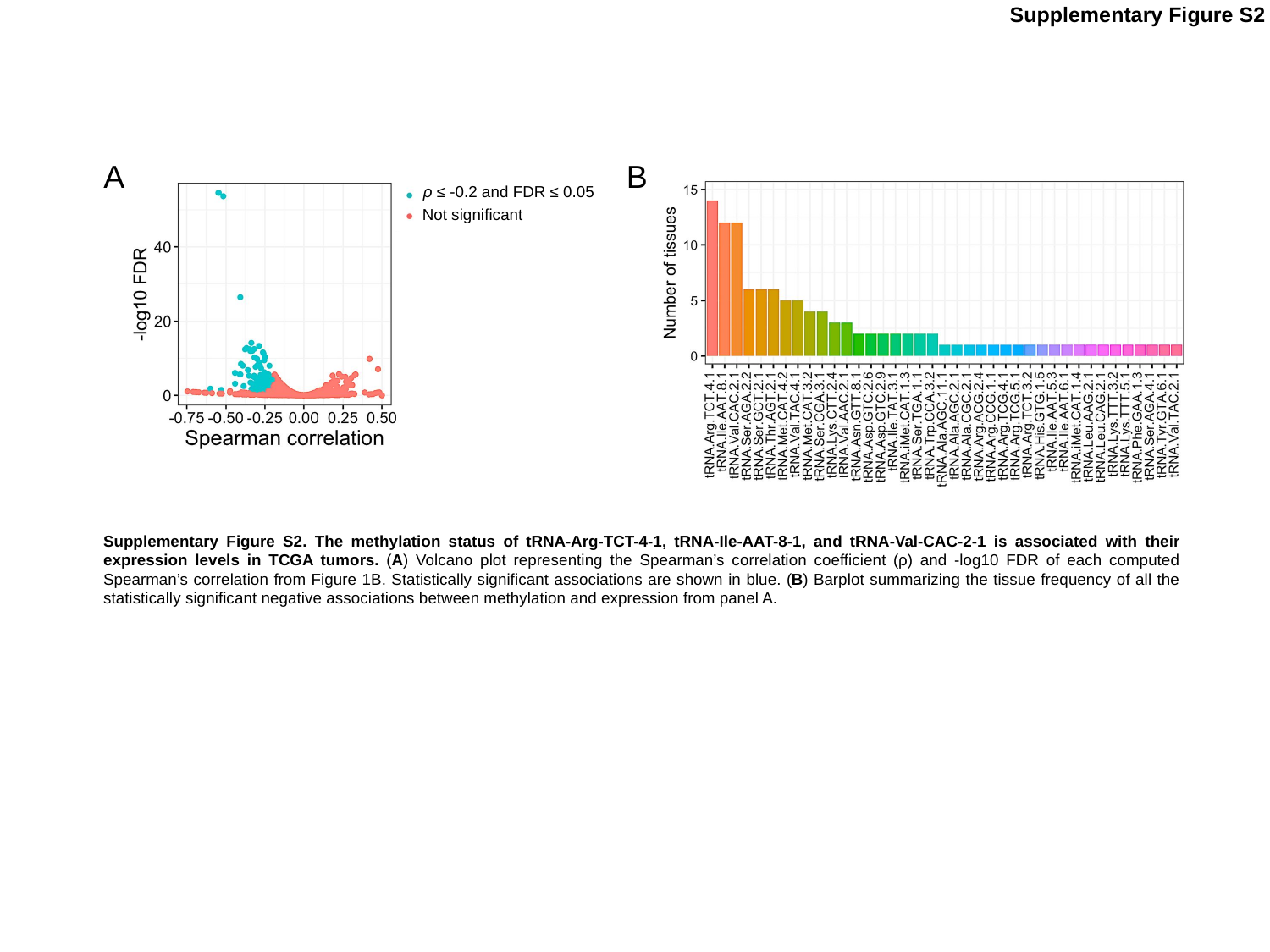

Supplementary Figure S2
A
B
ρ ≤ -0.2 and FDR ≤ 0.05
Not significant
Supplementary Figure S2. The methylation status of tRNA-Arg-TCT-4-1, tRNA-Ile-AAT-8-1, and tRNA-Val-CAC-2-1 is associated with their expression levels in TCGA tumors. (A) Volcano plot representing the Spearman’s correlation coefficient (ρ) and -log10 FDR of each computed Spearman’s correlation from Figure 1B. Statistically significant associations are shown in blue. (B) Barplot summarizing the tissue frequency of all the statistically significant negative associations between methylation and expression from panel A.
