# Supplementary material for: DNA methylation-associated dysregulation of transfer RNA expression in human cancer
Source: Mol Cancer. 2022 Feb 12;21:48. doi: 10.1186/s12943-022-01532-w (PMC8840503; doi:10.1186/s12943-022-01532-w)
Supplement: Supplementary file 3 — Additional file 3: Figure S3. Bisulfite genomic sequencing confirms the methylation status of tRNA-Arg-TCT-4-1 andtRNA-Ile-AAT-8-1 according to the HM450 microarray data. (A) DNA methylation status of tRNA-Arg-TCT-4-1 (cg12798524) and tRNA-Ile-AAT-8-1 (cg21339923) in DND41 and SW48 according to the HM450 microarray data. b-values corresponding to single CpGs are shown for each tDNA. (B) Bisulfite genomic sequencing of tRNA-Arg-TCT-4-1 (top) and tRNA-Ile-AAT-8-1 (below) genes in DND41 and SW48 cell lines. The tDNA gene genomic sequence is indicated with a blue bracket. The orange rectangles correspond to the A and B boxes of the tDNA. The TSS is marked with a black arrow. CpG dinucleotides are represented as short vertical lines, and their methylation status is denoted with black (methylated) or white (unmethylated) squares. The CpG included in the HM450 microarray is marked with a red asterisk. [file 12943_2022_1532_MOESM3_ESM.pptx]

## Slide 1
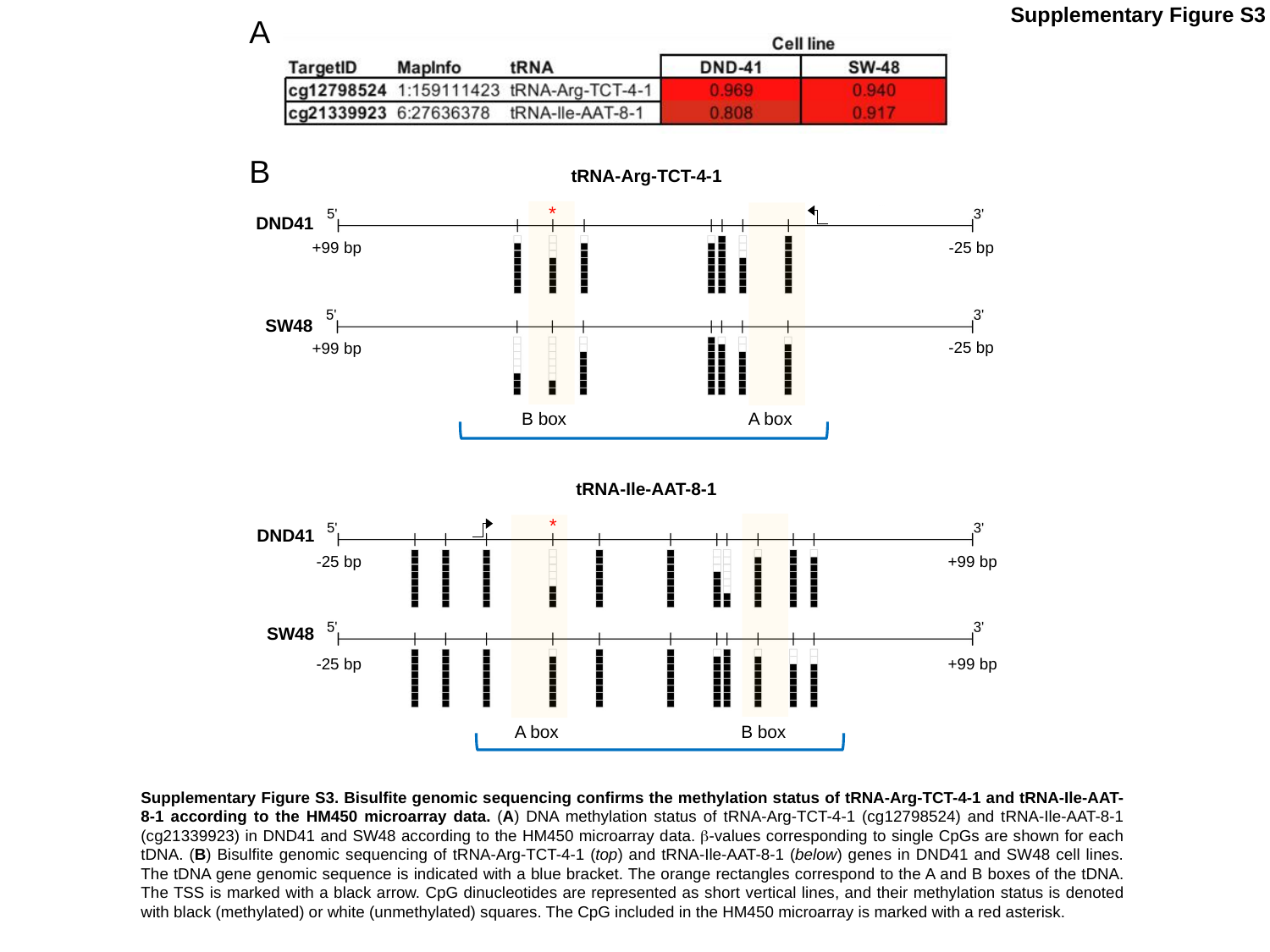

Supplementary Figure S3
A
B
tRNA-Arg-TCT-4-1
*
DND41
SW48
B box
A box
+99 bp
-25 bp
-25 bp
+99 bp
tRNA-Ile-AAT-8-1
*
DND41
SW48
A box
B box
-25 bp
+99 bp
-25 bp
+99 bp
Supplementary Figure S3. Bisulfite genomic sequencing confirms the methylation status of tRNA-Arg-TCT-4-1 and tRNA-Ile-AAT-8-1 according to the HM450 microarray data. (A) DNA methylation status of tRNA-Arg-TCT-4-1 (cg12798524) and tRNA-Ile-AAT-8-1 (cg21339923) in DND41 and SW48 according to the HM450 microarray data. β-values corresponding to single CpGs are shown for each tDNA. (B) Bisulfite genomic sequencing of tRNA-Arg-TCT-4-1 (top) and tRNA-Ile-AAT-8-1 (below) genes in DND41 and SW48 cell lines. The tDNA gene genomic sequence is indicated with a blue bracket. The orange rectangles correspond to the A and B boxes of the tDNA. The TSS is marked with a black arrow. CpG dinucleotides are represented as short vertical lines, and their methylation status is denoted with black (methylated) or white (unmethylated) squares. The CpG included in the HM450 microarray is marked with a red asterisk.
