# Supplementary material for: DNA methylation-associated dysregulation of transfer RNA expression in human cancer
Source: Mol Cancer. 2022 Feb 12;21:48. doi: 10.1186/s12943-022-01532-w (PMC8840503; doi:10.1186/s12943-022-01532-w)
Supplement: Supplementary file 4 — Additional file 4: Figure S4. Impact on growth and apoptosis of the DNA methylation inhibitor 5’-azacytidine(AZA) in DND41 and SW48 cancer cell lines. (A) MTT assay growth assay show a reduced growth of treated cells in both DND41 and SW48 cells lines upon the use of the demethylating agent. Data at each time points are the mean ± SD of four biological replicates. Statistical differences were determined using an unpaired two-tailed Student’s t-test at the 96 hours final time point. *** p < 0.001 (B) Apoptosis quantification by Annexin V incorporation reveals a significant percentage of cells affected by the treatment in DND41 and SW48 cell lines. Data shown represent the mean ± SD of four biological replicates analyzed by unpaired two-tailed Student’s t-test. * p < 0.05; *** p < 0.001. [file 12943_2022_1532_MOESM4_ESM.docx]

**SUPPLEMENTARY METHODS**

**Cell culture**

The T-acute lymphoblastic leukemia (T-ALL) cell line DND41 was purchased from the German Collection of Microorganisms and Cell Cultures (ACC-525). The colon adenocarcinoma SW48 cell line was obtained from the American Type Culture Collection (CCL-231). The endometrial carcinoma HEC1 cell line was acquired from the Japanese Collection of Research Bioresources Cell Bank (JCRB0042). DND41 was cultured in Roswell Park Memorial Institute (RPMI) 1640 medium. SW48 and HEC1 were cultured in Dulbecco’s Modified Eagle’s Medium (DMEM). All media were supplemented with 10% fetal bovine serum (FBS) and 1% penicillin/streptomycin, and cells were grown at 37°C and 5% CO_2_. All cell lines were authenticated by short tandem repeat profiling (LGS Standards SLU) and tested for the absence of mycoplasma. For DNA demethylating treatment, SW48 cells were treated with 1 µM 5-azacytidine (AZA) (Sigma, A2385) for a maximum of 96 hours, with medium renewal after 48 hours. DND41 cells were cultured with 0.5 µM AZA because of their increased sensitivity to this compound.

**DNA methylation analyses**

DNA methylation status of tDNA genes was determined by DNA methylation microarrays and bisulfite genomic sequencing. *In silico* DNA methylation analyses were carried out using the Infinium HumanMethylation450 (HM450) methylation microarray, which renders a 0-to-1 β-value to discern the level of methylation of the interrogated CpG. DNA methylation profiles from TCGA and a panel of approximately 1000 human cancer cell lines [1] were data mined to identify differentially methylated tDNA genes. Cross-reactive CpG were automatically discarded from the analysis [2]. tDNA were considered hypermethylated when the average β-value of the interrogated CpG dinucleotides was higher 0.33 in human tumors and normal samples from TCGA. For bisulfite genomic sequencing, 2 µg of genomic DNA were bisulfite-converted using the EZ DNA Methylation-Gold kit (Zymo Research, D5006) following the manufacturer’s indications. Bisulfite PCR primers were designed using MethylPrimer Express (Applied Biosystems) and are listed in **Supplementary Table S2**. Bisulfite PCR amplicons were cloned into the pGEMT-easy vector (Promega, AS1360) and transformed into competent bacteria. A minimum of 8 bacterial clones were subjected to Sanger sequencing to calculate methylation frequency. Results were analyzed with BioEdit software and methylated cytosines were mapped using BSMap software.

**Chromatin immunoprecipitation (ChIP)-qPCR**

Chromatin immunoprecipitation (ChIP) was conducted using the SimpleChip® Enzymatic Chromatin IP Kit (Cell Signaling, 9003) according to the manufacturer’s indications. A suspension of 2·10^7^ cells in 40 mL of culture media were crosslinked with 1% formaldehyde at room temperature for 10 minutes. The crosslink was quenched with the addition of 4 mL of 10X glycine solution for 5 minutes at room temperature. Then, cells were pelleted and washed twice with ice-cold PBS with protease inhibitor cocktail (PIC). The cell pellet was resuspended in 4 mL of 1X Buffer A with 0.5 µM dithiothreitol and PIC and incubated on ice 10 minutes. Nuclei were pelleted and washed with 4 mL of 1X Buffer B with 0.5 µM of dithiothreitol. Then, nuclei were resuspended with 400 µL of 1X Buffer B with 0.5 µM with 2 µL of micrococcal nuclease and incubated for 20 minutes at 37ºC with soft agitation. The digestion was stopped with 40 µL of 0.5 M of EDTA. The nuclei were pelleted, resuspended in 400 µL of 1X ChIP Buffer with PIC and sonicated to break the nuclear membranes. Nuclei lysates were clarified by centrifugation and the supernatant was recovered. 50 µL of this digested chromatin supernatant were used for chromatin digestion and concentration evaluation.

5 µg of digested chromatin were scaled to a final volume of 300 µL of 1X ChIP Buffer with PIC. 6 µL of it were removed and stored until further use as a 2% input control sample. The remaining sample was incubated overnight at 4ºC with rotation with 5 µL of the primary antibodies anti-GTF3C1 (Novus Biologicals, NB100-60657) or anti-POLR3A (Cell Signaling, 12825). Next day, samples were incubated with 20 µL of protein G magnetic beads at 4ºC with rotation for two hours to capture the chromatin fragments bound to the antibodies. Protein G beads were pelleted with a magnetic separation rack. Then, they were washed three times in 1 mL of ow salt buffer and once in 1 mL of high salt buffer at 4ºC during 5 minutes with rotation. After that, chromatin was eluted in 150 µL of 1X ChIP Elution Buffer at 65ºC for 30 minutes with vortexing followed by magnetic separation of the magnetic beads, when the supernatant was recovered. Finally, the IP and 2% input samples, in which 150 µL of 1X ChIP Elution buffer were added in advance, were incubated 2 hours at 65ºC with 6 µL of 5M NaCl and 2 µL proteinase K to reverse the crosslinking. The DNA was purified using the spin columns provided with the kit stored at -20ºC until further use.

qPCR was used to quantify the immunoprecipitated DNA. The qPCR reactions were conducted using 0.5 µL of chromatin sample, 4.5 µL of nuclease-free water, 4.85 µL of SYBR Green PCR Master Mix (Life Technologies, 4312704) and 0.15 µL of the appropriate primer mix at 10 µM. The fold change among samples was calculated as a percentage of the total chromatin following the formula 100·2^(CT adjusted input – CT sample)^. ChIP-qPCR primers are listed in **Supplementary Table S2**.

**Small RNA extraction and qRT-PCR**

tRNA expression in cancer cell lines was determined by qRT-PCR using commercially available isodecoder-specific primers. The small RNA fraction was extracted from cell lines by direct phenol acidic extraction. Fresh cell pellets were resuspended in 300 µL ice-cold resuspension buffer (0.3 M sodium acetate pH 4.5, 10 mM EDTA). 300 µL of cold acidic phenol:chloroform:isoamyl alcohol (125:24:1) pH 4.5 (Thermofisher Scientific, AM9722) were added. Samples were vigorously vortexed and centrifuged at maximum speed for 15 minutes at 4ºC. The aqueous phase was transferred to a new microcentrifuge tube containing 300 µL of acidic phenol:chloroform. The samples were again vortexed and centrifuged. The aqueous phase was transferred to a 15 mL tube containing 300 µL of acidic phenol:chloroform and 3.6 mL of absolute ethanol. After two hours of incubation on ice, samples were centrifuged and supernatant was discarded. The RNA pellets were washed twice in ethanol 80% and left to air-dry for 10 minutes. Then, they were resuspended in 90 µL of 10 mM sodium acetate pH 4.5 with 0.8 M LiCl. After centrifugation at maximum speed, supernatant containing the small RNA fraction was recovered. Next, it was precipitated with 10 µL of RNase-free 3 M sodium acetate pH 5.5 (Thermofisher Scientific, AM9740) and 300 µL of absolute ethanol for 40 minutes at -80ºC. Afterwards, samples were centrifuged at maximum speed for 30 minutes at 4ºC to precipitate the RNA. The RNA pellet was washed three times with ethanol 80% and vacuum-dried to eliminate all traces of ethanol. Finally, the RNA pellets were resuspended with 20 µL of RNase-free water.

1 µg al the small RNA fraction was retrotranscribed with the RevertAid First Strand cDNA Synthesis kit (Thermofisher Scientific, K1622) following manufacturer’s instructions. qRT-PCR were carried using 2.5 ng of cDNA in 5 µL, 4.85 µL of SYBR Green PCR Master Mix and 0.15 µL of the commercial primer mix at 10 µM. The fold change among samples was calculated following the ddCT formula using the expression U6 snRNA as an endogenous control. The references of the commercial primers as well as for U6 determination can be found in **Supplementary Table S2**.

**tDNA gene knockout**

tRNA-Arg-TCT-4-1 expression was abolished in the unmethylated HEC1 cell line using the CRISPR/Cas9 system as previously described [3]. Two sgRNA were designed using the online tool CHOPCHOP [4] to target the vicinity of tRNA-Arg-TCT-4-1 gene and were cloned into the pSpCas9(BB)-2A-GFP vector (Addgene, 48138). The two sgRNA were simultaneously transfected in HEC1 cells using JetPrime® Transfection Reagent (Polyplus transfections, 114-75). Green positive cell lines were isolated by cell sorting to establish clonal cell lines 48 hours after transfection and left for clonal expansion. Knockout clones were screened by amplification and sequencing of the sgRNA target region. sgRNA constructions and primers used for amplification of the targeted region are listed in **Supplementary Table S2**.

**Flow cytometry assays**

Cell cycle was analyzed by staining the cellular DNA content with propidium iodide. Briefly, a suspension of 10^7^ cells in 5 mL was fixed with 4.5 mL of ice-cold 70% ethanol for a time higher than 2 h. Fixed cells were washed with PBS and suspended in 1 mL of propidium iodide staining solution. Propidium iodide staining solution was prepared as follows: 200 µg of propidium iodide 2 mg of DNase-free RNase A were added to 10 mL of 0.1% (v/v) Triton X-100 in PBS. Samples were incubated 15 minutes at 37ºC and then were analyzed by flow cytometry. A minimum of 10,000 cells was analyzed per sample with a FACSCanto II (Beckton Dickinson). FlowJo software was used to quantify cell populations.

Apoptosis was analyzed by Annexin V staining. 10^6^ DND41 cells and 2·10^6^ SW48 cells were plated on 100 mm^2^ plates (10 mL/plate) and treated with AZA as described previously. Two time-points were taken (48h and 96h). In each time-point, cells were harvested, and concentration was calculated by trypan blue hemocytometer counting. 2·10^5^ cells were centrifuged and resuspended in 150 µL of Annexin-V binding buffer 1X (50 mL PBS pH 7.2 1X, 2.5 mM CaCl2, 140 mM NaCl, 10 mM HEPES) freshly supplemented with 0.01 µg/mL of Annexin-V-APC antibody (BioLegend, Ref: 640930). Cells were immediately analyzed in a BD FACSCanto II flow cytometer. FlowJo software was used to quantify cell populations.

**Cell proliferation determination**

Cell proliferation was measured by the sulforhodamine B (SRB) or the 3-(4,5-dimethylthiazol-2-yl)-2,5-diphenyltetrazolium bromide (MTT) assay. For the SRB assay, 500 cells were seeded in flat-bottomed 96-well plates and left for overnight adherence. In the appropriate time points, cell medium was removed from the plate and cells were fixed with 10% trichloroacetic acid for 1 hour at 4ºC. Then, they were washed with twice water and stained with 0.057% SRB in 1% acetic acid for 30 minutes at room temperature. After that, cells were washed twice with 1% acetic acid and finally resuspended in 100 µL of 10 mM Tris pH 10.0. Cell mass was assessed by measuring the absorbance at λ = 540 nm, and proliferation fold change was inferred by comparing the absorbance value to an initial value. For the MTT assay, 10,000 DND-41 cells and 2,000 SW-48 cells were plated in 96-well plates (100 µL/well). For each cell line, 8 replicates were treated with two doses (0h and 48h) of 5-aza-2’-deoxycytidine (AZA, 0.5 µM for DND-41 cells and 1 µM for SW-48 cells) and 8 replicates were left untreated. Four time-points were taken (0h, 48h, 72h and 96h). In each time-point, 10 µL of 5 mg/mL MTT (Sigma, M2128-10G) dissolved in PBS pH 7.2 1X (Gibco, Ref: 20012-019) were added to each well. After 3 hours of incubation at 37 ºC, 100 µL of lysis buffer (54% N,N-Dimethylformamide dissolved in H_2_O, 216 mg/L SDS, 2.7% acetic acid glacial, pH 4.6 adjusted with HCl) were added to each well and incubated 37 ºC overnight. Plates were analyzed in a ThermoScientific Multiskan Sky spectrophotometer at λ = 630 nm.

**Cell migration determination**

Cell migration capacity was assessed by the Transwell assay. 2·10^5^ cells were seeded in serum-free medium in the upper chamber of an 8 μm pore Transwell insert (Corning, 3422) and left for migration to the serum-containing lower chamber for 48 hours. Then, Transwell membranes were fixed with 10% trichloroacetic acid for 1 hour, washed with water and stained with 0.057% SRB in 1% acetic acid for 30 minutes. After washing the excess of SRB dye with 1% acetic acid, complete membrane pictures were taken, and ImageJ software was to calculate the percentage of membrane area occupied by cells to assess cell migration. Migration experiments were performed in triplicate.

**Data obtention and statistical analyses**

*In silico* tRNA expression was analyzed in TCGA samples by using the datasets generated by Zhang and coworkers that are available online [5]. Survival information of patients included in TCGA was obtained using the TCGAbiolinks Bioconductor R package [6]. The association between DNA methylation status and the correspondent tRNA expression in TCGA was assessed by Spearman’s correlation. Student’s t-test and Mann-Whitney U-test were used to compare differences between two groups as appropriate. Kaplan-Meier plots and logrank test, together with univariate Cox regression models, were used to estimate differences in overall survival probability. Statistical analyses were conducted within the R programming environment or with GraphPad Prism 5. Values of *p* < 0.05 were considered statistically significant. False discovery rate (FDR) method was used for multiple comparison *p*-values correction.

**Supplementary references**

1. Iorio F, Knijnenburg TA, Vis DJ, Bignell GR, Menden MP, Schubert M, et al. A Landscape of Pharmacogenomic Interactions in Cancer. Cell. 2016;166:740–54.

2. Chen YA, Lemire M, Choufani S, Butcher DT, Grafodatskaya D, Zanke BW, et al. Discovery of cross-reactive probes and polymorphic CpGs in the Illumina Infinium HumanMethylation450 microarray. Epigenetics. 2013;8:203–9.

3. Ran FA, Hsu PD, Wright J, Agarwala V, Scott DA, Zhang F. Genome engineering using the CRISPR-Cas9 system. Nat Protoc. 2013;8:2281–308.

4. Labun K, Montague TG, Gagnon JA, Thyme SB, Valen E. CHOPCHOP v2: a web tool for the next generation of CRISPR genome engineering. Nucleic Acids Res. 2016;44:W272–6.

5. Zhang Z, Ye Y, Gong J, Ruan H, Liu CJ, Xiang Y, et al. Global analysis of tRNA and translation factor expression reveals a dynamic landscape of translational regulation in human cancers. Commun Biol. 2018;1:234.

6. Colaprico A, Silva TC, Olsen C, Garofano L, Cava C, Garolini D, et al. TCGAbiolinks: An R/Bioconductor package for integrative analysis of TCGA data. Nucleic Acids Res. 2016;44:e71.
