# Supplementary material for: DNA methylation-associated dysregulation of transfer RNA expression in human cancer
Source: Mol Cancer. 2022 Feb 12;21:48. doi: 10.1186/s12943-022-01532-w (PMC8840503; doi:10.1186/s12943-022-01532-w)
Supplement: Supplementary file 5 — Additional file 5: Figure S5. The demethylation of tRNA-Arg-TCT-4-1 gene is associated with an increased tRNA expression in KIRP and UCEC TCGA tumors. (A) Frequency of tRNA-Arg-TCT-4-1 hypomethylation in tumors derived from TCGA according to the tissue of origin. (B) tRNA-Arg-TCT-4-1 expression is higher in those KIRP (top) and UCEC (below) TCGA tumors where this gene is hypomethylated. Statistical differences in tRNA expression between groups of samples were determined using a two-sided Mann-Whitney U-test; *** p < 0.001. Samples were considered hypomethylated or hypermethylated when tRNA-Arg-TCT-4-1 β-value was lower or higher than 0.33, respectively. [file 12943_2022_1532_MOESM5_ESM.pptx]

## Slide 1
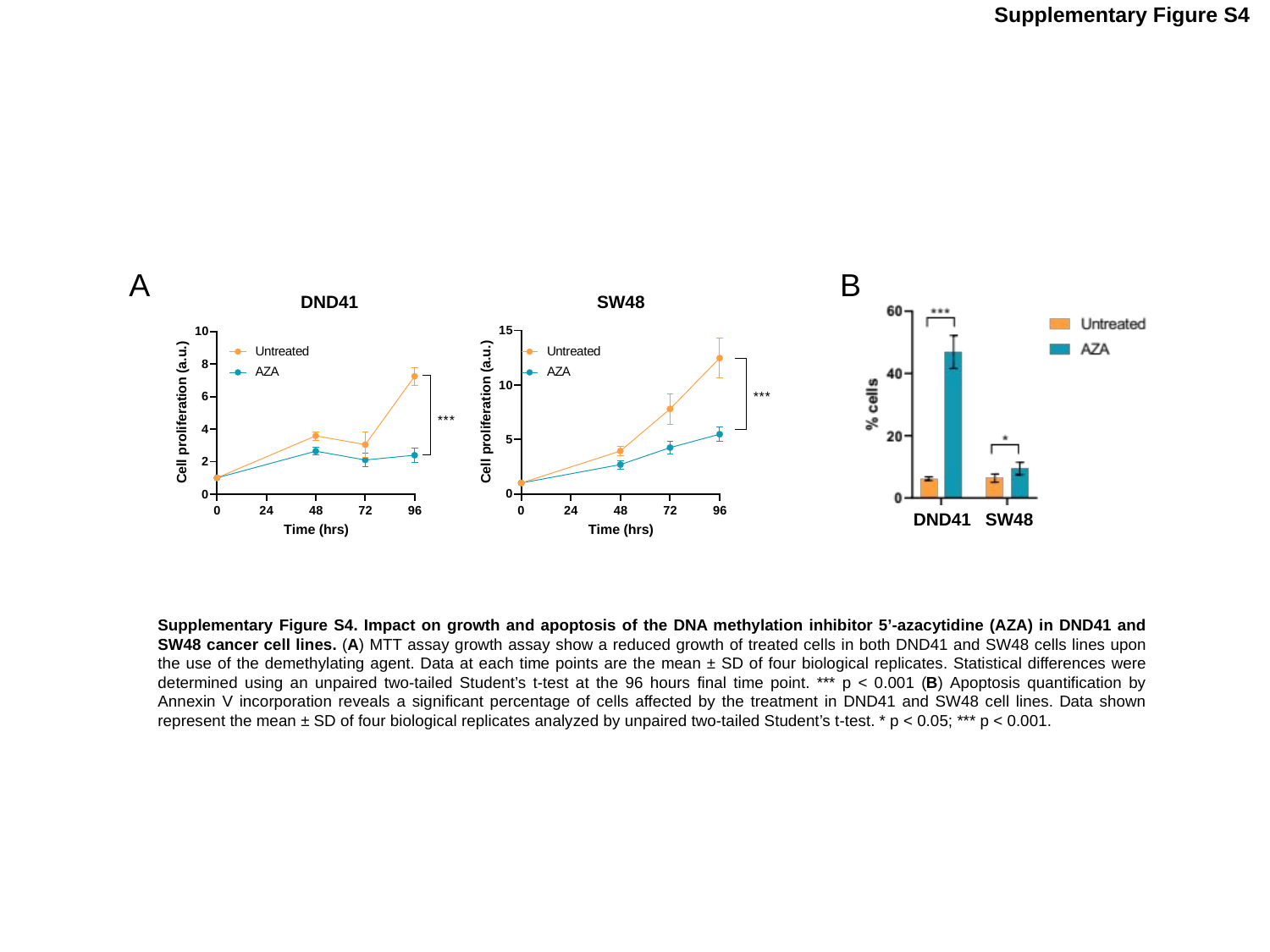

Supplementary Figure S4
A
B
SW48
DND41
DND41
SW48
Supplementary Figure S4. Impact on growth and apoptosis of the DNA methylation inhibitor 5’-azacytidine (AZA) in DND41 and SW48 cancer cell lines. (A) MTT assay growth assay show a reduced growth of treated cells in both DND41 and SW48 cells lines upon the use of the demethylating agent. Data at each time points are the mean ± SD of four biological replicates. Statistical differences were determined using an unpaired two-tailed Student’s t-test at the 96 hours final time point. *** p < 0.001 (B) Apoptosis quantification by Annexin V incorporation reveals a significant percentage of cells affected by the treatment in DND41 and SW48 cell lines. Data shown represent the mean ± SD of four biological replicates analyzed by unpaired two-tailed Student’s t-test. * p < 0.05; *** p < 0.001.
