# Supplementary material for: DNA methylation-associated dysregulation of transfer RNA expression in human cancer
Source: Mol Cancer. 2022 Feb 12;21:48. doi: 10.1186/s12943-022-01532-w (PMC8840503; doi:10.1186/s12943-022-01532-w)
Supplement: Supplementary file 7 — Additional file 7: Supplementary methods [file 12943_2022_1532_MOESM7_ESM.pptx]

## Slide 1
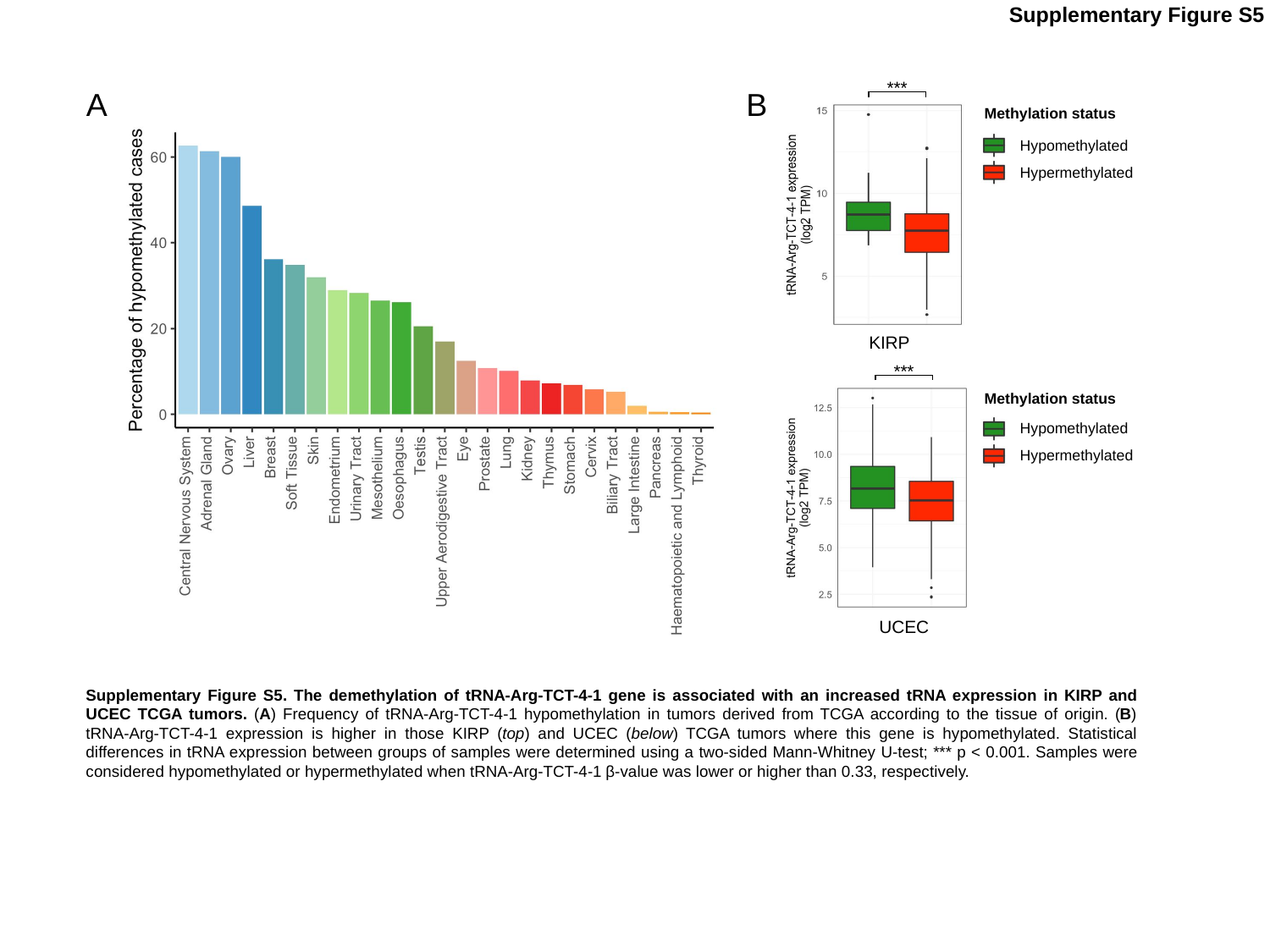

Supplementary Figure S5
***
KIRP
***
UCEC
A
B
Methylation status
Hypomethylated
Hypermethylated
Methylation status
Hypomethylated
Hypermethylated
Supplementary Figure S5. The demethylation of tRNA-Arg-TCT-4-1 gene is associated with an increased tRNA expression in KIRP and UCEC TCGA tumors. (A) Frequency of tRNA-Arg-TCT-4-1 hypomethylation in tumors derived from TCGA according to the tissue of origin. (B) tRNA-Arg-TCT-4-1 expression is higher in those KIRP (top) and UCEC (below) TCGA tumors where this gene is hypomethylated. Statistical differences in tRNA expression between groups of samples were determined using a two-sided Mann-Whitney U-test; *** p < 0.001. Samples were considered hypomethylated or hypermethylated when tRNA-Arg-TCT-4-1 β-value was lower or higher than 0.33, respectively.
