# Supplementary material for: DNA methylation-associated dysregulation of transfer RNA expression in human cancer
Source: Mol Cancer. 2022 Feb 12;21:48. doi: 10.1186/s12943-022-01532-w (PMC8840503; doi:10.1186/s12943-022-01532-w)
Supplement: Supplementary file 8 — Additional file 8: Table S1. Summary of the results obtained from the Univariate Cox regression analyses performed to compare TCGA patients’ prognosis according to their tDNA methylation levels. Blue-colored cells indicate statistical significance (FDR ≤ 0.05). [file 12943_2022_1532_MOESM8_ESM.pptx]

## Slide 1
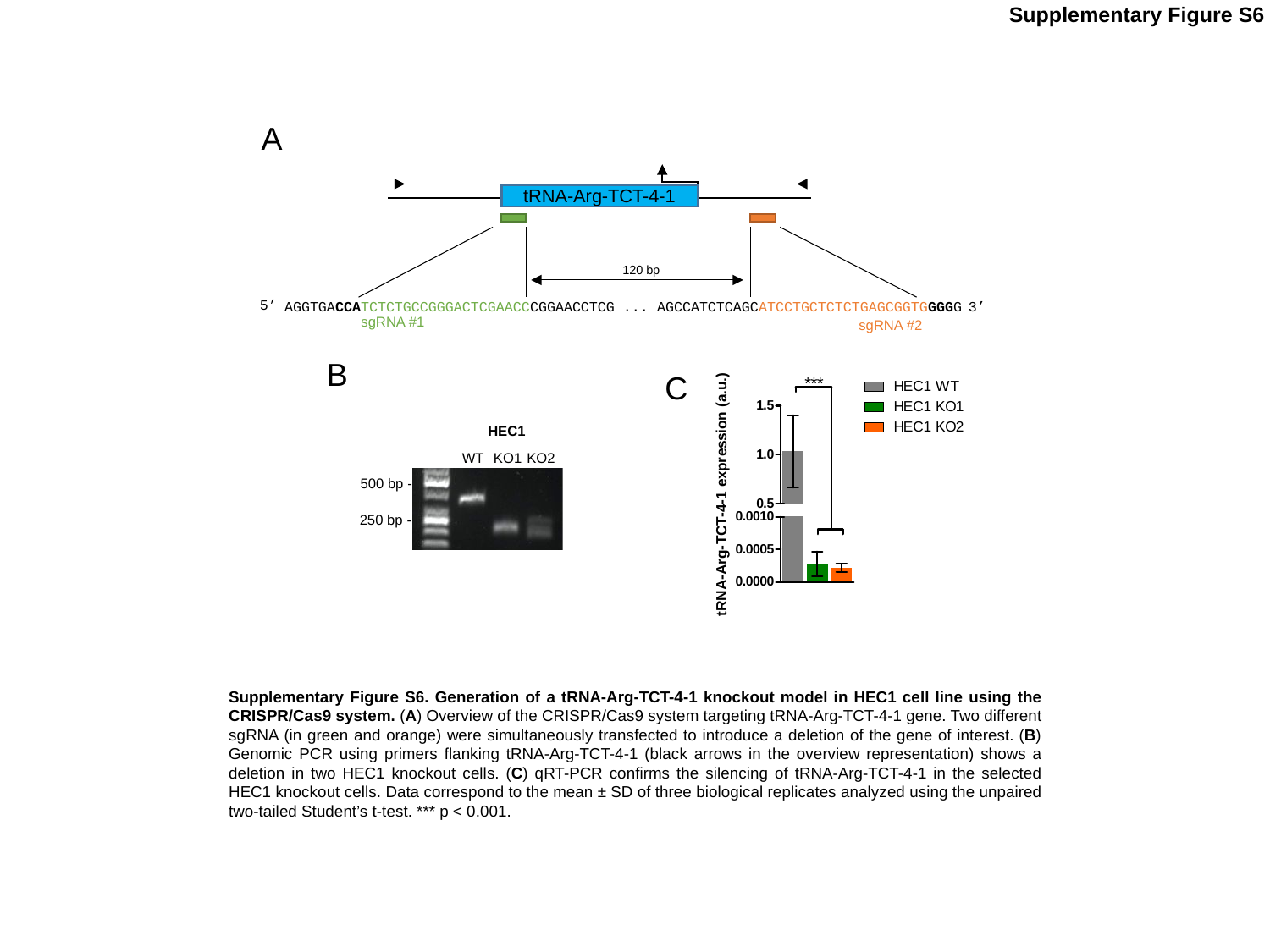

Supplementary Figure S6
A
tRNA-Arg-TCT-4-1
120 bp
5’
3’
AGGTGACCATCTCTGCCGGGACTCGAACCCGGAACCTCG ... AGCCATCTCAGCATCCTGCTCTCTGAGCGGTGGGGG
sgRNA #1
sgRNA #2
B
C
HEC1
WT
KO1
KO2
500 bp -
250 bp -
Supplementary Figure S6. Generation of a tRNA-Arg-TCT-4-1 knockout model in HEC1 cell line using the CRISPR/Cas9 system. (A) Overview of the CRISPR/Cas9 system targeting tRNA-Arg-TCT-4-1 gene. Two different sgRNA (in green and orange) were simultaneously transfected to introduce a deletion of the gene of interest. (B) Genomic PCR using primers flanking tRNA-Arg-TCT-4-1 (black arrows in the overview representation) shows a deletion in two HEC1 knockout cells. (C) qRT-PCR confirms the silencing of tRNA-Arg-TCT-4-1 in the selected HEC1 knockout cells. Data correspond to the mean ± SD of three biological replicates analyzed using the unpaired two-tailed Student’s t-test. *** p < 0.001.
