# Supplementary material for: DNA methylation-associated dysregulation of transfer RNA expression in human cancer
Source: Mol Cancer. 2022 Feb 12;21:48. doi: 10.1186/s12943-022-01532-w (PMC8840503; doi:10.1186/s12943-022-01532-w)
Supplement: Supplementary file 9 — Additional file 9: Table S2. Detailed list of primers used. All sequences are provided from 5’ to 3’. For tRNA qRT-PCR primers, the commercial reference is provided. [file 12943_2022_1532_MOESM9_ESM.docx]

**Supplementary Table S2.** Detailed list of primers used. All sequences are provided from 5’ to 3’. For tRNA qRT-PCR primers, the commercial reference is provided.

| ***Bisulfite genomic sequencing*** | | |
| --- | --- | --- |
| **tRNA-Arg-TCT-4-1** | Fd  Rv | ATTTTCCAACTATCCCTATCC  AGGATTTTTAAGGAAAAGGGTTTT |
| **tRNA-Ile-AAT-8-1** | Fd  Rv | ACCTATTCTTTTCATTTTTCACAATAA  GGTTTAGAGTTAAAATAGTTTGGATT |
| ***ChIP-qPCR*** | | |
| **tRNA-Arg-TCT-4-1** | Fd  Rv | ATTAGAAGTCCAGCGCGCTC  GATGGCTCGGTGATGCAGAA |
| **tRNA-Ile-AAT-8-1** | Fd  Rv | GTGTGGCCGGTTAGCTCA  GGTAAGTGAAGGGCCCCAC |
| ***qRT-PCR*** | | |
| **tRNA-Arg-TCT-4-1** | Arg-TCT-3 (Human) / AS-NR-001H-1-026 (ArrayStar) | |
| **tRNA-Ile-AAT-8-1** | Ile-AAT-5 (Human) / AS-NR-001H-1-080 (ArrayStar) | |
| **U6** | Fd  Rv | CTCGCTTCGGCAGCACA  AACGCTTCACGAATTTGCGT |
| ***CRISPR/Cas9 knockout*** | | |
| **sgRNA1** | Fd  Rv | CACCGTCTCTGCCGGGACTCGAACC  AAACGGTTCGAGTCCCGGCAGAGAC |
| **sgRNA2** | Fd  Rv | CACCGATCCTGCTCTCTGAGCGGTG  AAACCACCGCTCAGAGAGCAGGATC |
| **Sequencing** | Fd  Rv | TTCCTTTCTCTCCCCTATAGCC  ATCGGACCCAGAGTATTGAGAA |
